# Investigation on the mechanism of Shaoyao-Gancao Decoction in the treatment of gastric carcinoma based on network pharmacology and experimental verification

**DOI:** 10.18632/aging.204465

**Published:** 2023-01-03

**Authors:** Xin Zhou, Jiao Min, Mengying Che, Yating Yang, Yi Yang, Junfei Zhang, Lei Zhang, Xiaosha Zheng, Yan Chen, Ling Yuan, Yi Nan

**Affiliations:** 1 Key Laboratory of Ningxia Minority Medicine Modernization Ministry of Education, Ningxia Medical University, Yinchuan 750004, Ningxia Hui Autonomous Region, China; 2 Traditional Chinese Medicine College, Ningxia Medical University, Yinchuan 750004, Ningxia Hui Autonomous, China; 3 College of Pharmacy, Ningxia Medical University, Yinchuan 750004, Ningxia Hui Autonomous Region, China; 4 Clinical Medical College, Ningxia Medical University, Yinchuan 750004, Ningxia Hui Autonomous, China

**Keywords:** Shaoyao-Gancao Decoction, gastric carcinoma, network pharmacology, molecular docking, experimental verification

## Abstract

Background: Shaoyao-Gancao Decoction (SG-D) is a famous classical Chinese prescription that has been used in the treatment of numerous kinds of diseases. However, its mechanism of action in the treatment of Gastric carcinoma (GC) is not clear.

Methods: The active ingredients and targets of SG-D were screened using network pharmacology, and GC-related targets were retrieved through several databases. The protein-protein interaction network was then further constructed and GO and KEGG enrichment analysis were performed. Subsequently, molecular docking was carried out. Finally, we validated the results of the network pharmacology by performing *in vitro* cell experiments on CCK-8, apoptosis, cell cycle, platelet clone formation, and Western blotting with AGS cells.

Results: Three key active ingredients and 8 core targets were screened through a network pharmacological analysis, and the results of the KEGG indicated that the PI3K/Akt and MAPK signaling pathways are critical signaling pathways for SG-D to treat GC. Experimental results revealed that SG-D was able to inhibit AGS cells proliferation, induce apoptosis and arrest the cell cycle, and reduce the ability of cell clone formation by regulating the PI3K/Akt and MAPK signaling pathways.

Conclusions: Network pharmacology has shown that SG-D can act on multiple targets through multiple ingredients and treat GC by regulating multiple signaling pathways. *In vitro* cell experiments have also confirmed this, so as to provide a reference for subsequent related research.

## INTRODUCTION

Gastric carcinoma (GC) is a malignant tumor that originates from the gastric mucosa epithelial cells. According to the latest global cancer statistics, GC had the fifth highest global incidence rate and the fourth highest mortality rate in 2020 [1]. In the early stages of GC, there are no obvious symptoms, and clinical manifestations such as weight loss, epigastric pain, and loss of appetite can occur in the middle and late stages [2]. At present, the etiology of GC is not clear, and possible causative factors include Helicobacter pylori (HP) infection, geographical environment, diet, and genetics [3]. Due to the strong concealment of GC, surgery, radiotherapy, and chemotherapy are often ineffective, and tend to cause a range of side effects in the organism. Therefore, people are gradually turning their attention to Traditional Chinese Medicine (TCM).

In recent years, a growing number of studies have shown that certain Chinese herbal monomers and compounds have certain therapeutic effects on gastric carcinoma. Liang et al. [4] found that Galangin can inhibit the proliferation of GC cells by inhibiting NF-κb signaling pathway *in vitro* and *in vivo*. Berberine can induce apoptosis of GC cells, promote cell cycle arrest, inhibit cell invasion and migration by regulating the IL-6/JAK2/STAT3 signaling pathway, thus inhibiting the development of GC [5]. Li et al. [6] showed that Si-Jun-Zi decoction (SJZ) could inhibit the proliferation of GC cells by activating CMTM2, *in vivo* experiments also showed that SJZ could induce apoptosis of GC cells by down-regulating the mRNA expression level of p53 and Bcl-2 [7]. Banxia Xiexin Decoction (BXD) can significantly inhibit the cell viability and induce apoptosis of GC cells, which is mainly related to the inhibition of Wnt/β-catenin signaling pathway [8]. Through *in vivo* and *in vitro* experiments, Sun et al. [9] found that the combined application of Zuo Jin Wan (ZJW) and DDP could up-regulate the protein expression levels of Cleaved ROCK and P-PTEN, and down-regulate the expression levels of P-PI3K and p-cofilin-1 to induce apoptosis of GC cells. These results suggest that ZJW may inhibit DDP resistance in GC by regulating the ROCK/PTEN/PI3K signaling pathway. Some studies have found that Danggui Sini Decoction (DSD) can inhibit the Akt/Erk/p53 signaling pathway in a concentration-dependent manner and induce cell cycle arrest and apoptosis, thus inhibiting the proliferation of GC cells [10]. It can be seen that TCM plays an important role in the treatment of GC and is gradually attracting the attention of researchers. The ancient Chinese classical prescription for Shaoyao-Gancao Decoction (SG-D) is derived from Treatise on Febrile and Miscellaneous Diseases by Zhang Zhongjing, which consists of two kinds of Chinese herbal medicine, namely Shaoyao (*Paeonia lactiflora.*) and Gancao (*Glycyrrhiza uralensis* Fisch.). Shaoyao can preserve yin to nourish the blood, soften livers and relieve pain. Gancao can tonify middle-Jiao and Qi, relax spasm and relieve pain. Studies have shown that SG-D has a favorable therapeutic effect on digestive system diseases, such as anti-inflammation and protection of gastric mucosa, etc. [11]. However, the relation between SG-D and GC is still poorly studied.

**Figure 1:**
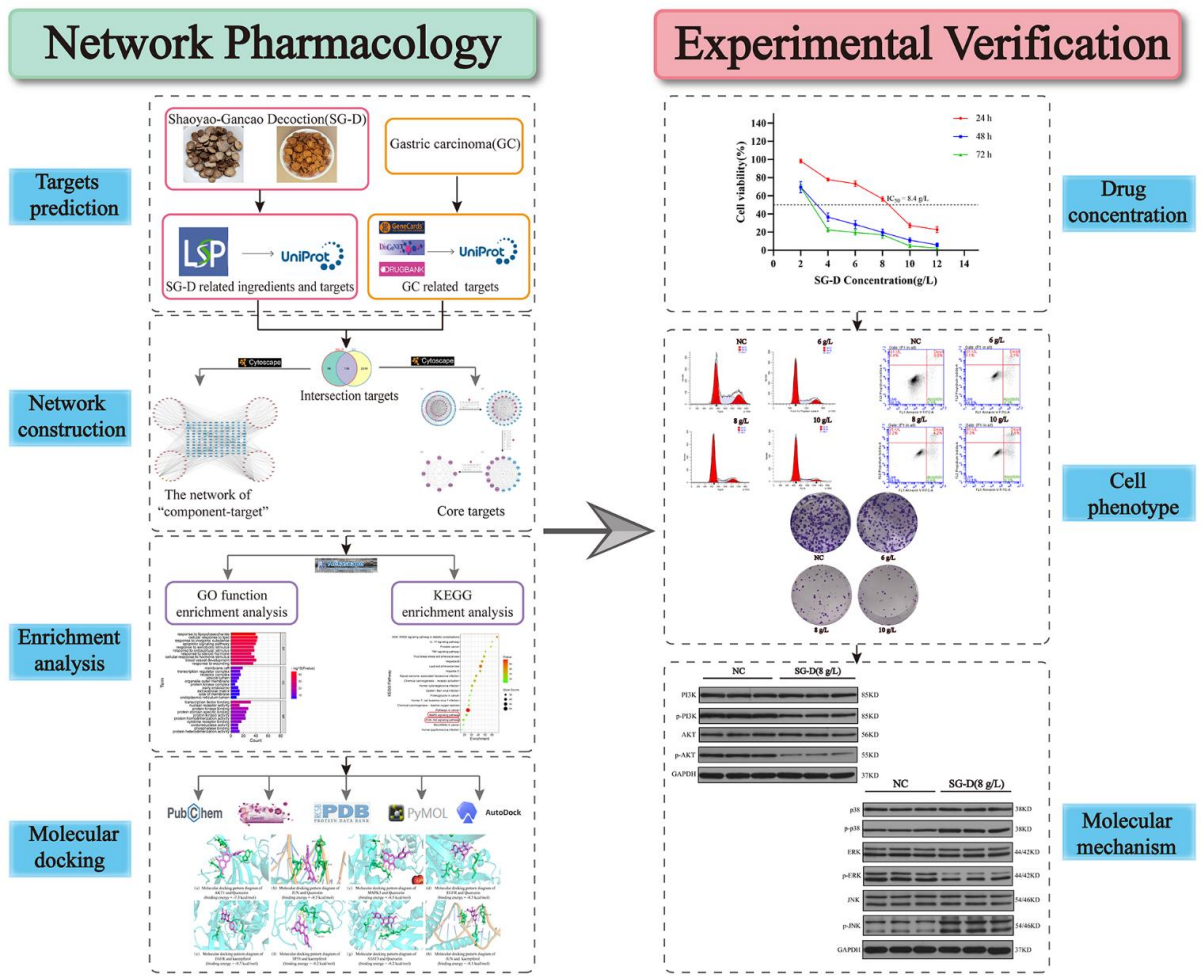
Flow chart.

In recent years, network pharmacology has been gradually applied in the field of TCM, which reveals the mechanism of drugs action based on the Chinese herbal medicine-components-targets-pathways-disease network [12]. This coincides with the characteristics of multi-components, multi-targets, and multi-pathways of TCM. Therefore, this research predicted the mechanism of action of SG-D in the treatment of GC through the method of network pharmacology and finally screened out the key active ingredients such as quercetin, core targets such as AKT, and signaling pathways such as PI3K/Akt and MAPK. Subsequently, the results were verified by *in vitro* cell experiments, which showed that SG-D was able to inhibit the proliferation of AGS cells, induce cell apoptosis and cycle arrest, and decrease the ability of cell clone formation by regulating PI3K/Akt and MAPK signaling pathways. Therefore, this study verified the efficacy of SG-D in the treatment of GC through the combination of network pharmacology and experimental verification, in order to provide a reference for future studies on SG-D in treating GC. The flow chart of this study is shown in Figure 1.

## RESULTS

### Active ingredients and targets for SG-D

**Figure 2:**
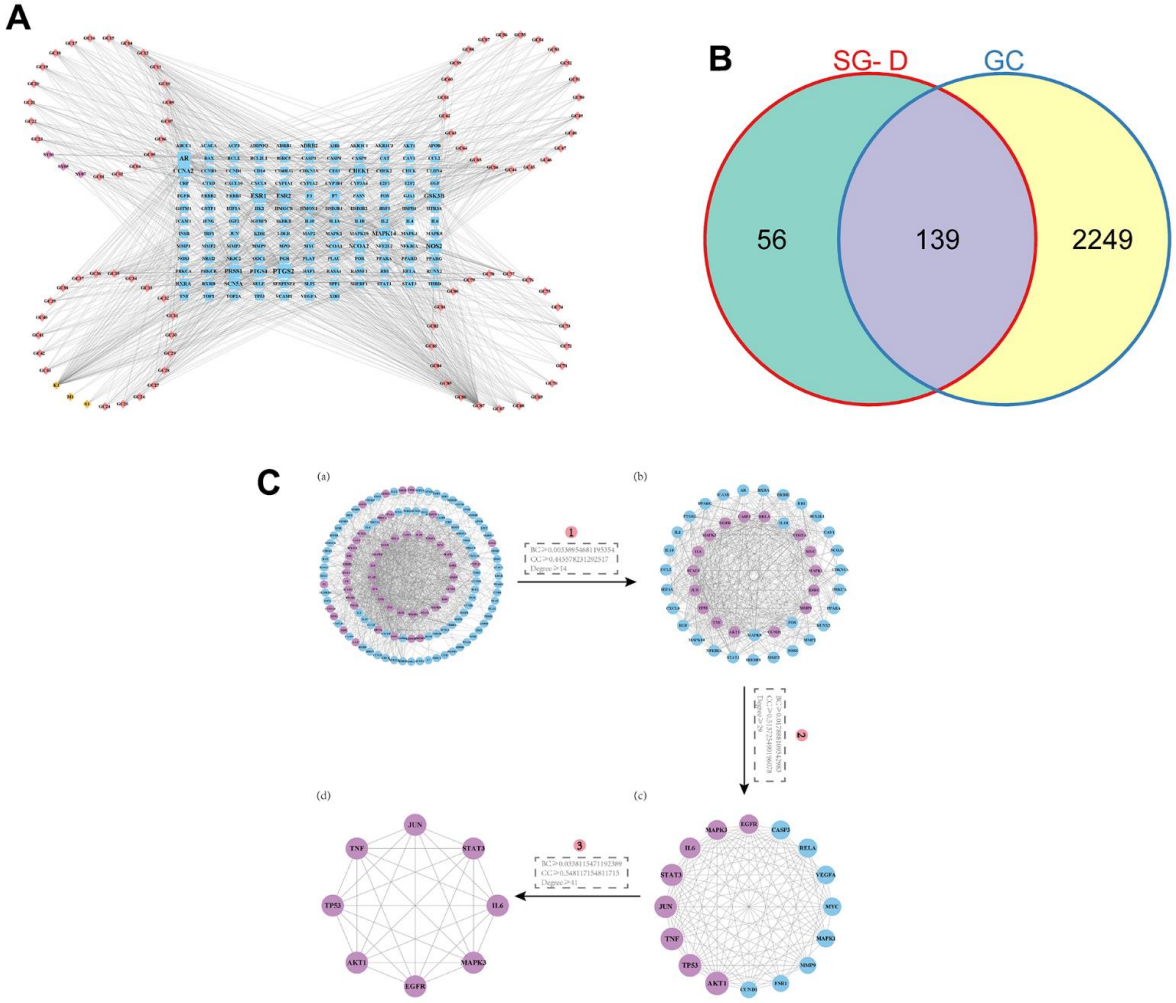
**Prediction of active ingredients and core targets.** (**A**) The “component-target” network. The active ingredients in Shaoyao are shown by the purple nodes, those in Gancao are represented by the pink nodes, the common active ingredients of SG-D by yellow nodes, and the common targets by blue node. The more active ingredients are connected to a node as it grows larger. (**B**) Venn diagram of intersecting targets of SG-D and GC. (**C**) The screening of core targets in the PPI network, (**a**) Containing 132 nodes and 1138 edges, purple nodes indicate targets with higher BC, CC, and Degree. (**b**) Containing 46 nodes and 491 edges. (**c**) Containing 16 nodes and 103 edges. (**d**) Containing 8 nodes and 28 edges.

After screening the active ingredients of SG-D with OB ≥ 30% and DL ≥ 0.18 in TCMSP database, a total of 105 active ingredients were obtained, including 13 from Shaoyao and 92 from Gancao (Supplementary Table 1 for details). At the same time, 1880 related targets were collected through the database, and after normalization processing by the Uniport database and removal of duplicates and non-human targets, a total of 195 targets for SG-D were obtained. Then, the Network Analyzer plug-in was used to calculate the Degree value of the nodes, and the active ingredients of Degree ranked in the top 20 were obtained (Table 1). Among them, quercetin, Kaempferol, and naringenin ranked high, with degrees of 104, 72, and 23, respectively. The construction of “component-target” network of SG-D is shown in Figure 2A, and the detailed names of ingredients are shown in Supplementary Table 2.

**Table 1:** Active ingredients information.

**Number**	**Molecule name**	**Degree**	**OB (%)**	**DL**	**Source**
1	quercetin	104	46.43	0.28	Gancao
2	kaempferol	72	41.88	0.24	Shaoyao/Gancao
3	naringenin	23	59.29	0.21	Gancao
4	1-Methoxyphaseollidin	20	69.98	0.64	Gancao
5	formononetin	19	69.67	0.21	Gancao
6	7-Methoxy-2-methyl isoflavone	18	42.56	0.2	Gancao
7	HMO	18	38.37	0.21	Gancao
8	Quercetin der.	18	46.45	0.33	Gancao
9	3’-Hydroxy-4’-O-Methylglabridin	18	43.71	0.57	Gancao
10	Jaranol	17	50.83	0.29	Gancao
11	licoisoflavanone	17	52.47	0.54	Gancao
12	3,22-Dihydroxy-11-oxo-delta(12)-oleanene -27-alpha-methoxycarbonyl-29-oic acid	17	34.32	0.55	Gancao
13	Isoglycyrol	17	44.7	0.84	Gancao
14	kanzonols W	16	50.48	0.52	Gancao
15	gadelaidic acid	16	30.7	0.2	Gancao
16	paeoniflorin	15	53.87	0.79	Shaoyao
17	Calycosin	15	47.75	0.24	Gancao
18	Glepidotin A	15	44.72	0.35	Gancao
19	Phaseolinisoflavan	15	32.01	0.45	Gancao
20	(2S)-7-hydroxy-2-(4-hydroxyphenyl)-8-(3-methylbut-2-enyl)chroman-4-one	15	36.57	0.32	Gancao

### Prediction of potential targets for SG-D in treating GC

2216 targets were obtained after screening and deleting duplicate entries in Genecards database, 321 targets were obtained after deleting duplicate entries in DisGeNET database with the marking criterion ≥ 0.3 and 73 targets were obtained after deleting duplicate entries in DrugBank database. Finally, 2388 GC-related targets were obtained by combining and deleting duplicate entries in the three databases. Then, 195 drug targets and 2388 disease targets were intersected to obtain 139 SG-D targets in the treatment of GC (Figure 2B).

### Analysis of the PPI network

A total of 139 intersection targets of SG-D and GC were uploaded into the STRING database, the confidence level was set to “> 0.700” and the free proteins were hidden, 139 nodes and 1138 edges were obtained after screening. Then, the results were imported into Cytoscape 3.9.0 software, and the topology of the PPI network was analyzed with the CytoNCA plug-in under the condition that BC, CC, and Degree were all greater than or equal to the median value, the first screening criteria were BC ≥ 0.00338954681195354, CC ≥ 0.445578231292517, Degree ≥ 14, the second screening criteria were BC ≥ 0.017888109542983, CC ≥ 0.513725490196078, Degree ≥ 29, and the third screening criteria were BC ≥ 0.0338115471192389, CC ≥ 0.548117154811715, Degree ≥ 41. Finally, the core network including 8 nodes and 28 edges were obtained. These targets were AKT1, TP53, TNF, JUN, STAT3, IL6, MAPK3 and EGFR, respectively (Figure 2C). We hypothesized that these targets are the key targets of SG-D in the treatment of GC.

### GO functional enrichment analysis and KEGG pathway enrichment analysis

**Figure 3:**
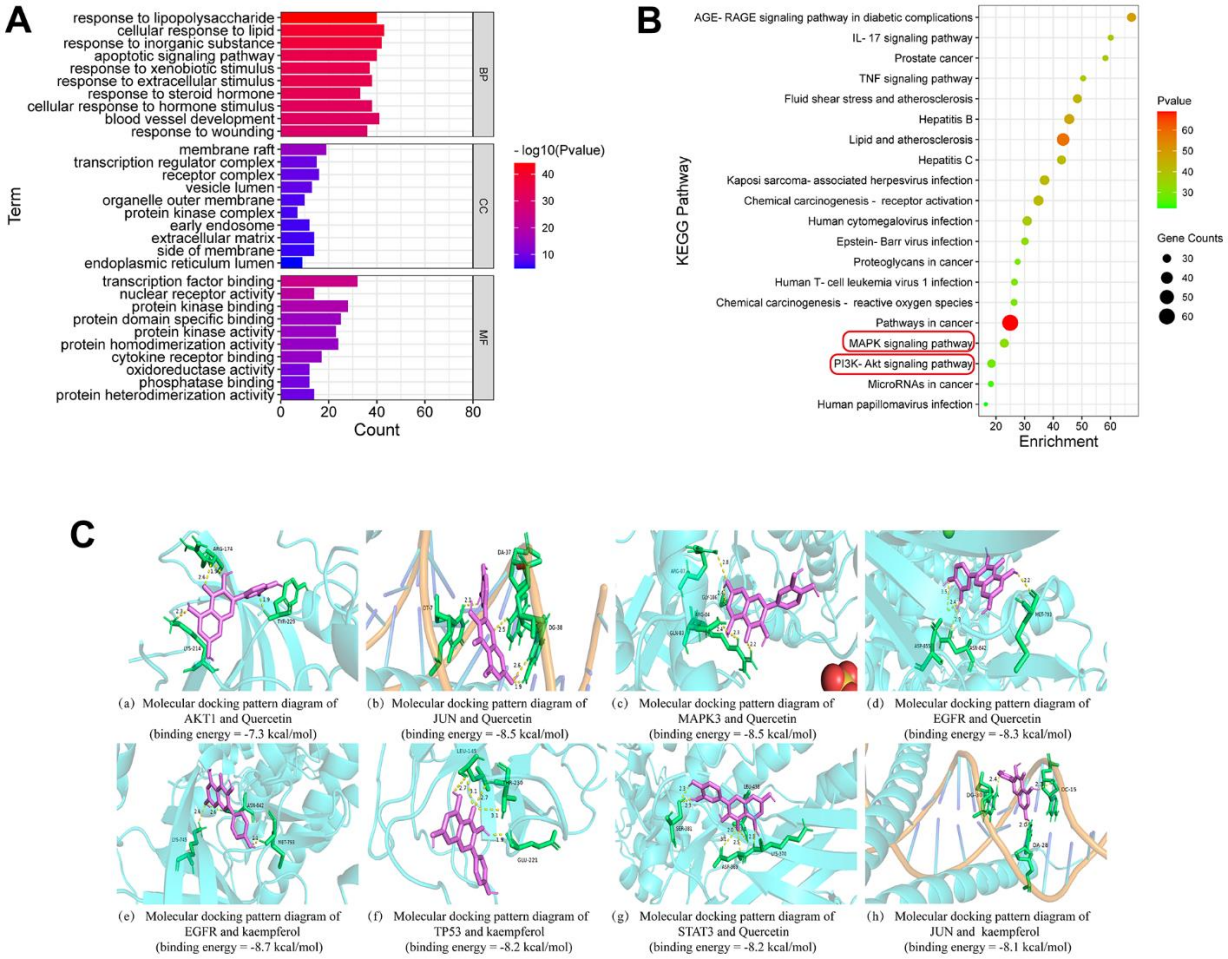
**Enrichment analysis and molecular docking.** (**A**) GO enrichment analysis. (**B**) The bubble diagram of KEGG pathway enrichment analysis. An increase in the value of P is shown by a change in the color of the nodes from green to red, and an increase in the number of genes is indicated by a change in the size of the nodes from tiny to large. (**C**) Molecular docking patterns of active ingredients and core targets.

The intersection targets were imported into the Metascape database for GO and KEGG enrichment analysis. The results of GO enrichment analysis showed that the therapeutic effect of SG-D on GC was mainly related to the biological processes such as response to lipopolysaccharide, cell response to lipid, response to inorganic substances, and apoptotic signaling pathway. The cellular components include membrane raft, transcriptional regulatory complex, and receptor complex. The molecular functions include transcription factor binding, protein kinase binding, and protein domain specific binding (Figure 3A). KEGG analysis enriched the top 20 most crucial pathways, and the results suggested that SG-D may exert anti-gastric carcinoma effects through pathways in cancer, MAPK signaling pathway and PI3K/Akt signaling pathway (Figure 3B).

### Molecular docking results

By using Auto Dock Vina software, the core targets were docked with the key active ingredients one by one to get the binding energy (Table 2). If the binding energy is less than - 4.25 kcal·mol^-1^, it indicates that the two have certain binding ability; if the binding energy is less than - 5.0 kcal·mol^-1^, the two have good binding ability; if the binding energy is less than - 7.0 kcal·mol^-1^, the binding ability of the two is particularly close. The molecular docking results showed that the 8 core targets have excellent binding energy with the key active ingredients, in agreement with network pharmacology results. The molecular docking pattern with higher binding energy are shown in Figure 3C.

**Table 2:** “Active ingredients-targets” binding energy.

**Uniprot Id**	**PDB ID**	**Gene symbol**	**Binding energy(kcal/mol)**		
			**Quercetin**	**Kaempferol**	**Naringenin**
P31749	3CQW	AKT1	-7.3	-6.4	-6.2
P01375	5M2I	TNF	-7.3	-7.1	-7.3
P04637	6GGB	TP53	-8.3	-8.2	-7.4
P05412	5T01	JUN	-8.5	-8.1	-7.4
P40763	6NJS	STAT3	-8.2	-8.0	-6.1
P05231	1ALU	IL6	-6.3	-6.7	-6.6
P27361	6GES	MAPK3	-8.5	-8.0	-7.3
P00533	6P1D	EGFR	-8.3	-8.7	-7.7

### SG-D decreased the viability of AGS cells SG-D

**Figure 4:**
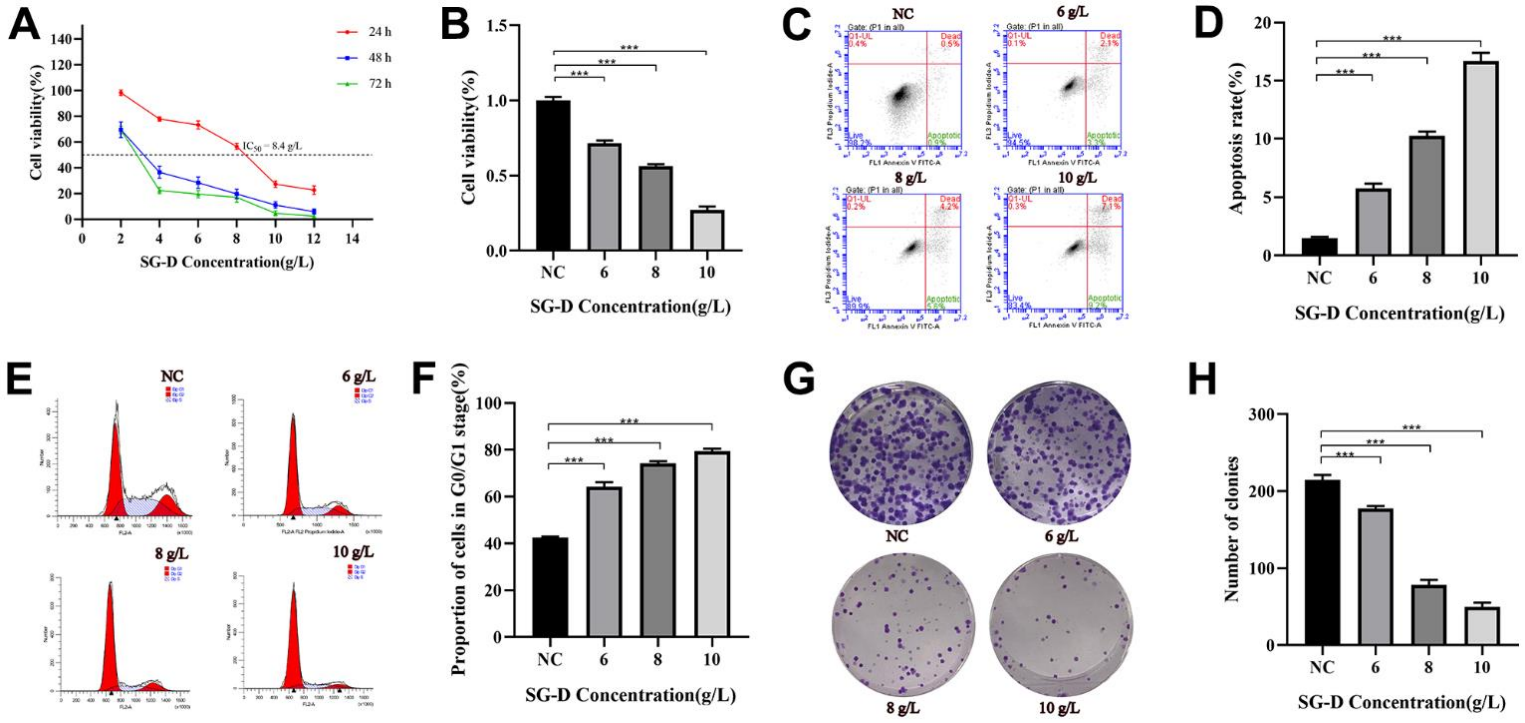
**The effect of SG-D on the phenotype of AGS cells.** (**A**) The impact of SG-D on the viability of AGS cells 24, 48, and 72 hours after treatment. (**B**) The impact of different SG-D concentrations (6, 8, and 10 g/L) on the viability of AGS cells. (**C**, **D**) Apoptosis rate. (**E**, **F**) Cell cycle distribution. (**G**, **H**) The quantity of generated cell clones. All experiments were repeated three times and the data were expressed as mean±SD, *p < 0.05, **p < 0.01, ***p < 0.001.

In order to detect the effect of SG-D on the proliferation of AGS cells, we treated human AGS cells with different concentrations of SG-D (2, 4, 6, 8, 10, and 12 g/L) for 24, 48, and 72 h. The results showed that SG-D could significantly decrease the viability of AGS cells in a concentration and time-dependent manner, that is, with the increase of drug concentration and treatment time, its inhibition gradually increased (Figure 4A). We found that cell proliferation was significantly inhibited and cell status was positive at 24 h of drug intervention, therefore we chose 24 h as the time for the follow-up drug intervention. When the concentration of SG-D was 8g/L, the inhibition rate of AGS cells was close to 50% (IC_50_ = 8.4 g/L for 24 h), so we chose the 8 g/L as the medium concentration and determined the low and high concentration in turn (Figure 4B).

### SG-D induced the apoptosis of AGS cells

The results of apoptosis showed that the apoptosis rates of AGS cells were (5.77 ± 0.40) %, (10.23 ± 0.38) %, (16.67 ± 0.72) % after intervention of low (6 g/L), middle (8 g/L) and high concentrations (10 g/L) of SG-D for 24 h, respectively, all higher than those of the control group (1.50 ± 0.10) % (Figure 4C, 4D).

### SG-D arrested AGS cells cycle in the G0/G1 phase

To further investigate the effect of SG-D on GC cells, we used Flow cytometry to examine the effect of SG-D on the cell cycle distribution of GC cells. The results showed that the percentage of AGS cells in G0/G1 phase changed significantly after treatment with different concentrations of SG-D (6, 8, and 10 g/L) for 24 h, which were (64.24 ± 1.97) %, (74.21 ± 0.95) % and (79.38 ± 1.09) %, respectively, they were all higher than the control group (42.50 ± 0.49) % (Figure 4E, 4F).

### SG-D inhibited the colony forming ability of AGS cells

In order to detect the effect of SG-D on the colony forming ability of AGS cells, we used SG-D (6, 8, and 10 g/L) to intervene cells. The experimental results showed that the number of cell clones formed in AGS cells was distinctly reduced after treatment with SG-D compared with the control group, and the inhibitory effect was more obvious with the increase of concentration (Figure 4G, 4H).

### SG-D inhibited AGS cells proliferation through PI3K/Akt and MAPK signaling pathways

**Figure 5:**
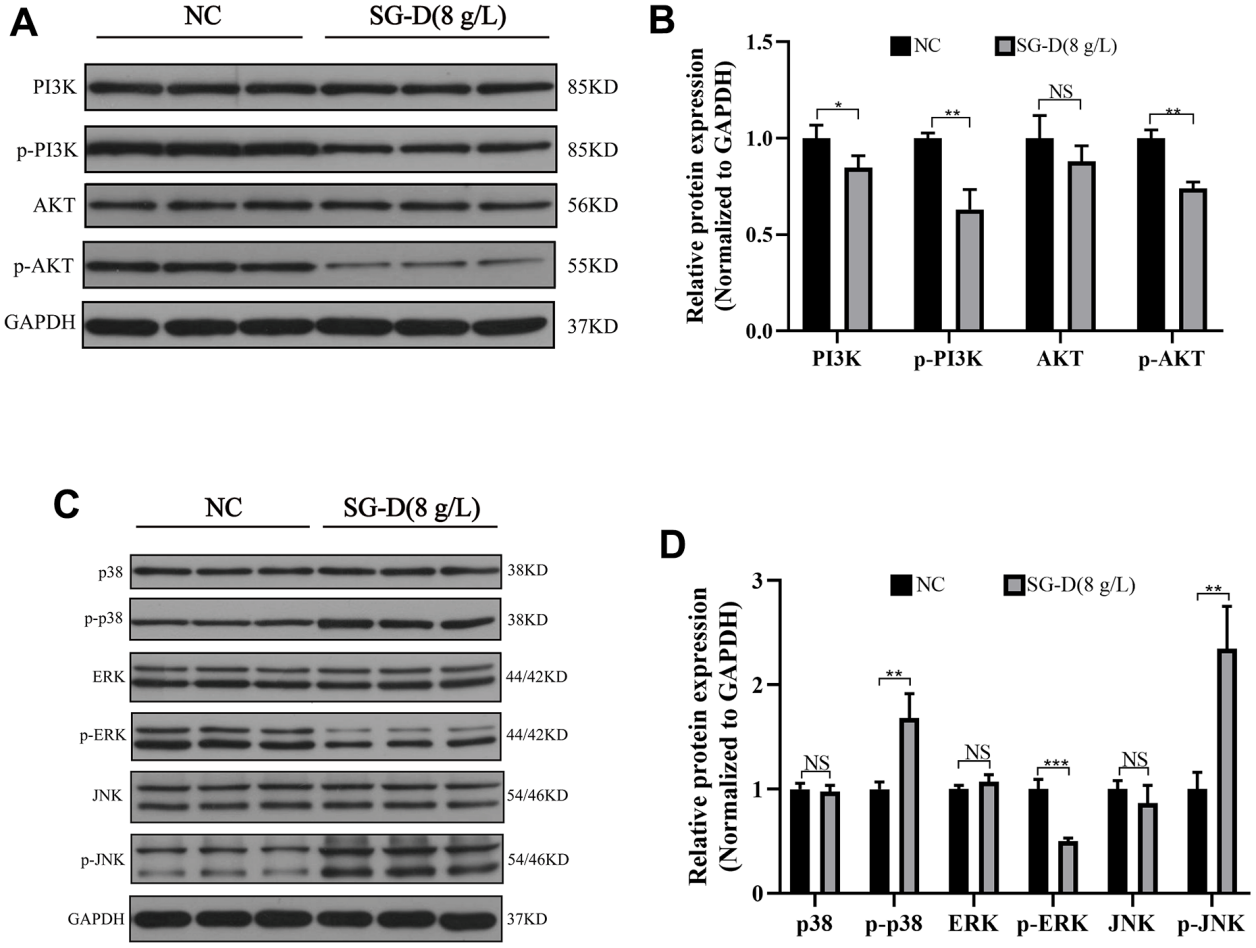
**The effect of SG-D on PI3K/Akt and MAPK pathways in AGS cells.** (**A**, **B**) The impact of SG-D on the expression of proteins connected to the PI3K/Akt signaling pathway in AGS cells. (**C**, **D**) The impact of SG-D on the expression of proteins connected to the MAPK signaling pathway in AGS cells. All experiments were repeated three times and the data were expressed as mean±SD, *p < 0.05, **p < 0.01, ***p < 0.001.

Based on the results of network pharmacology, we found that the PI3K/Akt and MAPK signaling pathways may be the vital pathways through which SG-D exerts its anti-gastric carcinoma effects. Therefore, we used Western blotting to examine PI3K, AKT, p38, JNK, ERK, and their corresponding phosphorylated products. The results showed that compared with the control group, SG-D had no significant effect on the total proteins of PI3K, AKT, p38, JNK, and ERK, but the phosphorylated products were changed to different degrees, SG-D down-regulated the protein expression levels of p-PI3K, p-AKT, p-ERK, and significantly up-regulated the protein expression levels of p-p38 and p-JNK (Figure 5A–5D).

## DISCUSSION

The occurrence and development of GC is an extremely complex process, which is generally characterized by abnormal cell proliferation, strong infiltration, and metastasis [13], and poses a great threat to human physical and mental health. At present, GC remains one of the leading causes of mortality in humans. Although the treatment of GC has been improved and the mortality has been reduced significantly, the prognosis of patients with GC is usually poor, and the side effects are more [14]. Therefore, it has become a new research direction to search for drugs that have anti-cancer effects and reduce side effects. SG-D, as a classical prescription lasting for thousands of years, has the therapeutic characteristics of “multi-ingredients, multi-targets, and multi-pathways”, which can regulate human body function and harmonize Yin and Yang. It has been widely used in the treatment of various diseases in recent years, but the related studies of GC have not been reported, and its mechanism of action is still unclear. As a result, this study reveals the mechanism of SG-D in the treatment of GC through network pharmacology and experimental verification.

The network pharmacology results showed that quercetin, kaempferol and naringin are the key active ingredients of SG-D in treating GC. Quercetin is a flavonoid compound with various pharmacological effects such as anti-inflammatory, antioxidant, anti-cancer and immunomodulatory effects [15]. Studies have found that quercetin inhibits the proliferation of GC cells by inhibiting HER2 kinase [16], apoptosis can also be induced by regulating the expression of apoptosis-related proteins [17]. Kaempferol is also a flavonoid compound and has anti-inflammatory, antioxidant, and anti-cancer effects [18]. Kaempferol has been shown to be involved in a variety of biological processes, including cell proliferation [19], apoptosis [20], cell cycle [20] and autophagy [21]. Naringenin is a dihydroflavonoid compound that has anti-cancer, anti-inflammatory, and anti-oxidant pharmacological activities [22]. It can down-regulate the protein expression levels of MMP2, MMP9, BCL-2, and Survivin, and up-regulate the protein expression levels of Bax and cleaved Caspase-3 to attenuates cell invasion and migration ability and induce apoptosis, thereby exerting anti-cancer effects [23]. According to the results of PPI network analysis, we screened out several core targets of SG-D in the treatment of GC, such as AKT1, TNF, EGFR, MAPK3, STAT3, IL6, TP53, and JUN. These targets are closely related to cell proliferation, cell cycle, cell apoptosis, and other biological processes, and play a key role in the treatment of GC with SG-D. GO function enrichment analysis revealed that the therapeutic effect of SG-D on GC is mainly related to biological processes and molecular functions such as cell apoptosis and protein kinase binding. KEGG enrichment analysis has shown that the PI3K/Akt and MAPK signaling pathways are essential pathways for SG-D to treat GC. The results of molecular docking also showed that the key ingredients can be tightly bound to the core targets. Therefore, based on the results of network pharmacology, we suggest that SG-D has a therapeutic effect on GC cells by regulating PI3K/Akt and MAPK signaling pathways.

To further clarify the molecular mechanism of SG-D in the treatment of GC, we verified the predicted results by *in vitro* cell experiments. We used AGS cells to conduct the CCK-8 experiment, and the results showed that the cell viability of AGS cells decreased after 24 h of intervention with SG-D, and the cell viability decreased more significantly with the increase of drug concentration and the extension of time, which indicated that SG-D had a significant inhibitory effect on AGS cells in a concentration-and time-dependent manner. The change in the cell cycle plays an influential role in the occurrence and development of tumors [24]. Our experiment results showed that compared with the control group, the number of cells in G0/G1 phase increased with the increase of SG-D concentration [from (64.24 ± 1.97) % to (79.38 ± 1.09) %], which suggested that SG-D could arrest the AGS cells cycle in G0/G1 phase, thus inhibiting cell proliferation. Apoptosis is a death mechanism tightly regulated by multiple apoptotic genes [25]. Our results showed that compared with the control group, the apoptosis rate increased with the increase of drug concentration [from (1.50 ± 0.10) % to (16.67 ± 0.72) %], which indicated that SG-D could significantly induce apoptosis in AGS cells in a concentration- dependent manner. We also examined the effect of SG-D on the proliferative ability of AGS cells by plate clone formation assay, and the results showed that the number of clonogenic cells was significantly reduced, which indicated that SG-D could significantly inhibit the colony forming ability of AGS cells, and this is consistent with the results of our previous experiments. The PI3K/Akt signaling pathway is closely related to the occurrence and development of tumors, and it is commonly found to be highly expressed in GC. Phosphatidylinositol 3-kinase (PI3K) is an intracellular phosphatidylinositol kinase that can be classified as class I, class II, and class III PI3K depending on the structure and substrate [26]. Relevant studies have shown that class I PI3K plays a crucial role in the occurrence and development of tumors, and participates in cell proliferation, survival, and other processes [27]. Class I PI3K is a heterodimer made up of the regulatory subunit p110 and the catalytic subunit p85 [28]. The activated PI3K catalyzes the phosphorylation of Phosphatidylinositol (4, 5) bisphosphate (PIP2) and conversion to Phosphatidylinositol (3, 4, 5) triphosphate (PIP3) [29]. The AKT1 gene encodes a serine/threonine protein kinase, which consists of AKT1, AKT2, and AKT3 and is an important downstream signaling molecule of PI3K [30]. PIP3 binds to AKT, translocates it to the cell membrane, thus, phosphorylated AKT is activated and subsequently involved in a variety of cellular functions [31]. Mitogen-activated protein kinase (MAPK) is a group of highly conserved protein kinases [32]. The MAPK signaling pathway is one of the hubs of multiple signaling pathways involved in cell proliferation, apoptosis, and inflammation [33]. Previous studies have confirmed that the MAPK signaling pathway is extensively involved in various cellular activities of GC cells [34]. Among the four major MAPK signaling pathways, ERK, JNK, p38/MAPK, and ERK5 [35], ERK1/2 is the most extensively studied and is primarily involved in cell proliferation and differentiation [36]. After extracellular stimulation, ERK1/2 is activated and phosphorylated, and then transferred from the cytoplasm to the nucleus to phosphorylate substrates such as P90 ribosomal S6 kinase (P90rsk), proto-oncogene c-fos and c-jun, thus participating in cell proliferation, survival and other processes [37]. Apoptosis is mainly associated with JNK and p38 [38]. While JNK is activated by various extracellular stimuli such as cytokines and growth factors and then participates in cell activities [39], while p38 is activated by DNA damage and oxidative stress, thus regulating various cellular functions [40]. In this study, we examined the effect of SG-D on the PI3K/Akt and MAPK signaling pathways in AGS cells by Western Blot. The results showed that SG-D down-regulated the protein expression levels of p-PI3K, p-AKT, and p-ERK and up-regulated the protein expression of p-p38 and p-JNK, which indicated that SG-D can inhibit the proliferation of AGS cells by inhibiting the PI3K/Akt signaling pathway and the ERK pathway in the MAPK signaling pathway, and activating the p38 and JNK pathways.

In summary, this study showed that SG-D can induce apoptosis of GC cells, promote cell cycle arrest and reduce cell clone formation ability by regulating PI3K/Akt and MAPK signaling pathways, thus inhibiting the proliferation of GC cells, which could provide a scientific basis for the related research of SG-D in the treatment of GC. However, our study is limited to *in vitro* cell experiments due to time and funding issues, and additional experiments are needed to support our future research. Therefore, we will continue to explore the link between SG-D and GC in the future with the following study. First, the therapeutic effect of SG-D on GC is investigated by *in vitro* animal experiments. Second, to explore the effect of SG-D in reducing chemotherapeutic drug sensitivity in combination with chemotherapy drugs. Third, the molecular mechanism of SG-D in GC treatment through gene silencing, co-IP, EMSA, and other methods should be further investigated.

## CONCLUSIONS

In this study, we investigated the molecular mechanism of SG-D in the treatment of GC through network pharmacology and experimental verification. The results showed that SG-D could inhibit the proliferation of GC cells by regulating PI3K/Akt and MAPK signaling pathways, induced apoptosis and cell cycle arrest, and reduced colony forming ability. Our results confirm the reliability of network pharmacology analysis and provide a strong scientific basis for further research.

## MATERIALS AND METHODS

### Screening of active ingredients and corresponding targets for SG-D

The active ingredients of Shaoyao and Gancao were collected by Traditional Chinese Medicine Systems Pharmacology Database and Analysis Platform (TCMSP, https://old.tcmsp-e.com/tcmsp.php) [41], and were screened on the conditions of oral bioavailability (OB) ≥ 30% and drug likeness (DL) ≥ 0.18 [42]. The corresponding targets were collected by the TCMSP database, and the results were standardized in UniProt database (UniProtKB, https://www.uniprot.org/), the filter criteria were “Reviewed” and “Human”. Subsequently, the active ingredients of SG-D and their co-action targets were imported into Cytoscape3.9.0 software, and the node degree values were calculated using the Network Analyzer plug-in, then the key active ingredients were screened out and the component-target Network was constructed.

### Screening of GC targets and intersecting targets

In the GeneCards database (https://www.genecards.org/) [43], DisGeNET database (https://www.disgenet.org/) [44] and DrugBank database (https://go.drugbank.com/) [45], “Gastricc carcinoma”, “Cancer of the stomach”, “Stomach cancer”, “Gastric cancer” and “Stomach neoplasm” were used as keywords to search for targets related to GC. The target information from the three databases were combined and the duplicated items were deleted to finally obtain the GC targets. Then, using Uniprot database, the targets of SG-D and GC were transformed into the corresponding Gene Symbol and mapped into Venn diagram (http://www.bioinformatics.com.cn/) [46], so as to obtain the intersection targets of SG-D and GC.

### Construction of the PPI Network

Imported the intersecting targets into the STRING database (https://cn.string-db.org/), and the filtering criteria was set as “Homo sapiens” and “combined score > 0.700” [47]. The screened data were imported into Cytoscape 3.9.0 software, and the network topology analysis was performed using CytoNCA plug-in under the condition of BetweennessCentrality (BC), ClosenessCentrality (CC), and Degree were all greater than or equal to the median value [48]. The core genes of SG-D in GC treatment were subsequently obtained.

### Enrichment analysis of GO and KEGG pathways

The Metascape database (https://metascape.org/) [49] was used for Gene Ontology (GO) analysis and Kyoto Encyclopedia of Genes and Genomes (KEGG) analysis of intersection targets, and species were set as “H.sapiens”, the screening threshold was “Min Overlap = 3”, “Pvalue Cutoff < 0.01” and “Min Enrichment = 1.5” [50]. The top 20 items of P value in KEGG enrichment analysis were selected for analysis, and the bubble diagram of the KEGG pathway was generated using bioinformatics (http://www.bioinformatics.com.cn/).

### Molecular docking

Using the PubChem database (https://pubchem.ncbi.nlm.nih.gov/) [51], download the key active ingredients of the 2D structures, then import them into Chem3D software to convert and optimize them into 3D structures. Download the 3D structures of core targets from RCSB PDB database (https://www.rcsb.org/), set species as “Homo sapiens”, and imported them into PyMOL software for processing. Then ligands and receptors were processed by AutoDockTools1.5.6 software, and AutoDockVina was used for molecular docking, the results were visualized using PyMOL software [52].

### Experimental materials

The human gastric adenocarcinoma AGS cells were purchased from Shanghai Genechem Co., Ltd. (CAT: GCC-ST0003RT, Shanghai, China), the cells have been identified by STR without cross contamination. Fetal bovine serum was purchased from Gemini (CAT: 900108, Shanghai, China). DME/F-12 medium was purchased from Hyclone (CAT: SH30023.01, USA). Cell Counting Kit-8 Kit was purchased from Dojindo (CAT: CK04, Japan). Jiangsu KeyGEN Bio TECH Corp., Ltd. (CAT: KGA512/KGA107, Jiangsu, China) provided the Cell Cycle Detection Kit and the Annexin-V FITC/PI Double Staining Apoptosis Detection Kit. Crystal Violet was purchased from Beijing Biotopped Technology Co., Ltd. (CAT: C6470, Beijing, China). Immunoway provided PI3K, P-PI3K, AKT, p-AKT, p38, p-p38, p-JNK, and GADPH antibodies, while CST provided Erk, p-ERK, and JNK antibodies.

SG-D formula granules came from the Yinchuan Hospital, Affiliated Hospital of Traditional Chinese Medicine of Ningxia Medical University, Shaoyao (Paeonia lactiflora.) and Gancao (Glycyrrhiza uralensis Fisch.) are produced from Inner Mongolia and Xinjiang, China, respectively, and were prepared in a 1:1 ratio [53]. The equivalent formula granules were dissolved in 15 ml ddH2O to prepare SG-D solution (Shaoyao: 1 g granule = 5 g crude drugs; Gancao: 1 g granule = 3 g crude drugs). After centrifugation at 12000 rpm for 10 min, we collected the supernatant and filtered it with a 0.22 μm needle filter. Finally, the solution concentration was calculated as 200 g/L based on the crude drug content.

### Cell culture

AGS cells were cultured in DME/F-12 medium containing 10% FBS and 1% penicillin (100 u/ml)/streptomycin (100 μg/ml) and placed in an environment of 37° C and 5 % CO_2_, cell passage cultivation every 2-3 days.

### Cell viability assay

AGS cells were seeded into 96-well plates at the density of 6 × 10^3^ cells per well. The experiment was divided into three groups: the blank control group, the negative control group, and the drug intervention group. The cells were treated with different concentrations of SG-D (2, 4, 6, 8, 10, and 12 g/L) for 24, 48, 72 hours, respectively. Then, 10 μl of CCK-8 solution was added to each well and cultured in the incubator for 2 hours. The optical density (OD) value of each group of cells at 450 nm wavelength was measured with a microplate reader, each group had 3 replicates. Cell viability = [(experimental group absorbance-blank group absorbance)]/[(control group absorbance-blank group absorbance)] × 100%.

### Apoptosis assay

The cells were seeded in 6-well plates, and when the cell confluence rate of cells ranged from 70% to 80%, supernatants were taken and continued to be cultured with drug-containing medium, providing 3 replicates per group. After 24 hours, the cells were digested and collected, washed the cells twice with pre-cooled PBS, and 500 μl Binding Buffer, 5 μl Annexin V-FITC and 5 μl Propidium Iodide were added, then reacted in a dark place for 5~15 min and flow cytometry was then used for detection and analysis within 1 hour.

### Cell cycle assay

The cells in decent growth conditions were seeded in 6-well plates and cultured for a period of time, after which different concentrations of drug-containing medium were added, and 3 replicates were established in each group. After 24 hours of drug intervention, cells were collected, washed with PBS, and immobilized at 4° C for 2 hours until overnight. After that, the stationary liquid was washed with PBS, and 500 μl of working solution (RNase A:PI = 1:9) was added into each well and reacted in a dark place for 30~60 min at room temperature. Finally, flow cytometry was used to detect the percentage of cells per cycle.

### Plate clone formation assay

Based on the cell count results, 500 cells per well were seeded into 6-well plates, and 3 replicates were set up. Conventional culture was carried out for 10-14 days, changing the medium every three days, and culture was terminated when the visible clones were formed. Later, the cells were washed with PBS, and 1 ml 4% paraformaldehyde was added and fixed at 4° C for 30 min, then added 500 μl crystal violet solution and stained for 15 min at room temperature, and then washed the staining fluid, took photos after dried at room temperature, statistical analysis based on clone sizes (Diameter > 1 mm).

### Western blot analysis

Cells from each group were collected, and the total protein was extracted by adding the configured lysate (The ratio was 10 μl of phosphatase inhibitor, 1 μl of Protease inhibitor, and 5 μl of 100 mM PMSF to each 1 ml of cold Lysis Buffer). The protein content was determined using the BCA Protein Quantitation Kit, and the protein was then denaturated. At the end of SDS-PAGE, target proteins were transferred to PVDF membranes, sealed with 5% skim milk, washed several times with TBST, and then incubated overnight at 4° C with primary antibodies. The PVDF membrane was then cleaned with TBST several times, the corresponding secondary antibodies were added and incubated at room temperature for 1 h. Finally, ECL luminescent solution and an ultra-sensitive multifunctional imager were used for detection, and gray value analysis of protein bands was performed using ImageJ software (NIH, Bethesda, MD, USA).

### Statistical analysis

All data were statistically analyzed with GraphPad prism 8 software and expressed as mean ± SD. Using one-way ANOVA and t-tests analyzed the statistical differences, *p* < 0.05 indicated a significant difference.

## Supplementary Material

Supplementary Table 1

Supplementary Table 2
