# Supplementary material for: Investigation on the mechanism of Shaoyao-Gancao Decoction in the treatment of gastric carcinoma based on network pharmacology and experimental verification
Source: Aging (Albany NY). 2023 Jan 3;15(1):148–63. doi: 10.18632/aging.204465 (PMC9876642; doi:10.18632/aging.204465)
Supplement: Supplementary Table 1 [file aging-15-204465-s001.docx]

**Supplementary Table 1. Active ingredients of SG-D.**

| **Number** | **Source** | **Mol ID** | **Molecule Name** | **OB (%)** | **DL** |
| --- | --- | --- | --- | --- | --- |
| SY01 | Shaoyao | MOL001930 | benzoyl paeoniflorin | 31.27 | 0.75 |
| SY02 | Shaoyao | MOL000359 | sitosterol | 36.91 | 0.75 |
| SY03 | Shaoyao | MOL000358 | beta-sitosterol | 36.91 | 0.75 |
| SY04 | Shaoyao | MOL000422 | kaempferol | 41.88 | 0.24 |
| SY05 | Shaoyao | MOL001919 | (3S,5R,8R,9R,10S,14S)-3,17-dihydroxy-4,4,8,10,14-pentamethyl-2,3,5,6,7,9-hexahydro-1H-cyclopenta[a]phenanthrene-15,16-dione | 43.56 | 0.53 |
| SY06 | Shaoyao | MOL001921 | Lactiflorin | 49.12 | 0.8 |
| SY07 | Shaoyao | MOL001924 | paeoniflorin | 53.87 | 0.79 |
| SY08 | Shaoyao | MOL000492 | (+)-catechin | 54.83 | 0.24 |
| SY09 | Shaoyao | MOL000211 | Mairin | 55.38 | 0.78 |
| SY10 | Shaoyao | MOL001910 | 11alpha,12alpha-epoxy-3beta-23-dihydroxy-30-norolean-20-en-28,12beta-olide | 64.77 | 0.38 |
| SY11 | Shaoyao | MOL001928 | albiflorin_qt | 66.64 | 0.33 |
| SY12 | Shaoyao | MOL001925 | paeoniflorin_qt | 68.18 | 0.4 |
| SY13 | Shaoyao | MOL001918 | paeoniflorgenone | 87.59 | 0.37 |
| GC01 | Gancao | MOL001484 | Inermine | 75.18 | 0.54 |
| GC02 | Gancao | MOL001792 | DFV | 32.76 | 0.18 |
| GC03 | Gancao | MOL000211 | Mairin | 55.38 | 0.78 |
| GC04 | Gancao | MOL002311 | Glycyrol | 90.78 | 0.67 |
| GC05 | Gancao | MOL000239 | Jaranol | 50.83 | 0.29 |
| GC06 | Gancao | MOL002565 | Medicarpin | 49.22 | 0.34 |
| GC07 | Gancao | MOL000354 | isorhamnetin | 49.6 | 0.31 |
| GC08 | Gancao | MOL000359 | sitosterol | 36.91 | 0.75 |
| GC09 | Gancao | MOL003656 | Lupiwighteone | 51.64 | 0.37 |
| GC10 | Gancao | MOL003896 | 7-Methoxy-2-methyl isoflavone | 42.56 | 0.2 |
| GC11 | Gancao | MOL000392 | formononetin | 69.67 | 0.21 |
| GC12 | Gancao | MOL000417 | Calycosin | 47.75 | 0.24 |
| GC13 | Gancao | MOL000422 | kaempferol | 41.88 | 0.24 |
| GC14 | Gancao | MOL004328 | naringenin | 59.29 | 0.21 |
| GC15 | Gancao | MOL004805 | (2S)-2-[4-hydroxy-3-(3-methylbut-2-enyl)phenyl]-8,8-dimethyl-2,3-dihydropyrano[2,3-f]chromen-4-one | 31.79 | 0.72 |
| GC16 | Gancao | MOL004806 | euchrenone | 30.29 | 0.57 |
| GC17 | Gancao | MOL004808 | glyasperin B | 65.22 | 0.44 |
| GC18 | Gancao | MOL004810 | glyasperin F | 75.84 | 0.54 |
| GC19 | Gancao | MOL004811 | Glyasperin C | 45.56 | 0.4 |
| GC20 | Gancao | MOL004814 | Isotrifoliol | 31.94 | 0.42 |
| GC21 | Gancao | MOL004815 | (E)-1-(2,4-dihydroxyphenyl)-3-(2,2-dimethylchromen-6-yl)prop-2-en-1-one | 39.62 | 0.35 |
| GC22 | Gancao | MOL004820 | kanzonols W | 50.48 | 0.52 |
| GC23 | Gancao | MOL004824 | (2S)-6-(2,4-dihydroxyphenyl)-2-(2-hydroxypropan-2-yl)-4-methoxy-2,3-dihydrofuro[3,2-g]chromen-7-one | 60.25 | 0.63 |
| GC24 | Gancao | MOL004827 | Semilicoisoflavone B | 48.78 | 0.55 |
| GC25 | Gancao | MOL004828 | Glepidotin A | 44.72 | 0.35 |
| GC26 | Gancao | MOL004829 | Glepidotin B | 64.46 | 0.34 |
| GC27 | Gancao | MOL004833 | Phaseolinisoflavan | 32.01 | 0.45 |
| GC28 | Gancao | MOL004835 | Glypallichalcone | 61.6 | 0.19 |
| GC29 | Gancao | MOL004838 | 8-(6-hydroxy-2-benzofuranyl)-2,2-dimethyl-5-chromenol | 58.44 | 0.38 |
| GC30 | Gancao | MOL004841 | Licochalcone B | 76.76 | 0.19 |
| GC31 | Gancao | MOL004848 | licochalcone G | 49.25 | 0.32 |
| GC32 | Gancao | MOL004849 | 3-(2,4-dihydroxyphenyl)-8-(1,1-dimethylprop-2-enyl)-7-hydroxy-5-methoxy-coumarin | 59.62 | 0.43 |
| GC33 | Gancao | MOL004855 | Licoricone | 63.58 | 0.47 |
| GC34 | Gancao | MOL004856 | Gancaonin A | 51.08 | 0.4 |
| GC35 | Gancao | MOL004857 | Gancaonin B | 48.79 | 0.45 |
| GC36 | Gancao | MOL004860 | licorice glycoside E | 32.89 | 0.27 |
| GC37 | Gancao | MOL004863 | 3-(3,4-dihydroxyphenyl)-5,7-dihydroxy-8-(3-methylbut-2-enyl)chromone | 66.37 | 0.41 |
| GC38 | Gancao | MOL004864 | 5,7-dihydroxy-3-(4-methoxyphenyl)-8-(3-methylbut-2-enyl)chromone | 30.49 | 0.41 |
| GC39 | Gancao | MOL004866 | 2-(3,4-dihydroxyphenyl)-5,7-dihydroxy-6-(3-methylbut-2-enyl)chromone | 44.15 | 0.41 |
| GC40 | Gancao | MOL004879 | Glycyrin | 52.61 | 0.47 |
| GC41 | Gancao | MOL004882 | Licocoumarone | 33.21 | 0.36 |
| GC42 | Gancao | MOL004883 | Licoisoflavone | 41.61 | 0.42 |
| GC43 | Gancao | MOL004884 | Licoisoflavone B | 38.93 | 0.55 |
| GC44 | Gancao | MOL004885 | licoisoflavanone | 52.47 | 0.54 |
| GC45 | Gancao | MOL004891 | shinpterocarpin | 80.3 | 0.73 |
| GC46 | Gancao | MOL004898 | (E)-3-[3,4-dihydroxy-5-(3-methylbut-2-enyl)phenyl]-1-(2,4-dihydroxyphenyl)prop-2-en-1-one | 46.27 | 0.31 |
| GC47 | Gancao | MOL004903 | liquiritin | 65.69 | 0.74 |
| GC48 | Gancao | MOL004904 | licopyranocoumarin | 80.36 | 0.65 |
| GC49 | Gancao | MOL004905 | 3,22-Dihydroxy-11-oxo-delta(12)-oleanene-27-alpha-methoxycarbonyl-29-oic acid | 34.32 | 0.55 |
| GC50 | Gancao | MOL004907 | Glyzaglabrin | 61.07 | 0.35 |
| GC51 | Gancao | MOL004908 | Glabridin | 53.25 | 0.47 |
| GC52 | Gancao | MOL004910 | Glabranin | 52.9 | 0.31 |
| GC53 | Gancao | MOL004911 | Glabrene | 46.27 | 0.44 |
| GC54 | Gancao | MOL004912 | Glabrone | 52.51 | 0.5 |
| GC55 | Gancao | MOL004913 | 1,3-dihydroxy-9-methoxy-6-benzofurano[3,2-c]chromenone | 48.14 | 0.43 |
| GC56 | Gancao | MOL004914 | 1,3-dihydroxy-8,9-dimethoxy-6-benzofurano[3,2-c]chromenone | 62.9 | 0.53 |
| GC57 | Gancao | MOL004915 | Eurycarpin A | 43.28 | 0.37 |
| GC58 | Gancao | MOL004917 | glycyroside | 37.25 | 0.79 |
| GC59 | Gancao | MOL004924 | (-)-Medicocarpin | 40.99 | 0.95 |
| GC60 | Gancao | MOL004935 | Sigmoidin-B | 34.88 | 0.41 |
| GC61 | Gancao | MOL004941 | (2R)-7-hydroxy-2-(4-hydroxyphenyl)chroman-4-one | 71.12 | 0.18 |
| GC62 | Gancao | MOL004945 | (2S)-7-hydroxy-2-(4-hydroxyphenyl)-8-(3-methylbut-2-enyl)chroman-4-one | 36.57 | 0.32 |
| GC63 | Gancao | MOL004948 | Isoglycyrol | 44.7 | 0.84 |
| GC64 | Gancao | MOL004949 | Isolicoflavonol | 45.17 | 0.42 |
| GC65 | Gancao | MOL004957 | HMO | 38.37 | 0.21 |
| GC66 | Gancao | MOL004959 | 1-Methoxyphaseollidin | 69.98 | 0.64 |
| GC67 | Gancao | MOL004961 | Quercetin der. | 46.45 | 0.33 |
| GC68 | Gancao | MOL004966 | 3'-Hydroxy-4'-O-Methylglabridin | 43.71 | 0.57 |
| GC69 | Gancao | MOL000497 | licochalcone a | 40.79 | 0.29 |
| GC70 | Gancao | MOL004974 | 3'-Methoxyglabridin | 46.16 | 0.57 |
| GC71 | Gancao | MOL004978 | 2-[(3R)-8,8-dimethyl-3,4-dihydro-2H-pyrano[6,5-f]chromen-3-yl]-5-methoxyphenol | 36.21 | 0.52 |
| GC72 | Gancao | MOL004980 | Inflacoumarin A | 39.71 | 0.33 |
| GC73 | Gancao | MOL004985 | icos-5-enoic acid | 30.7 | 0.2 |
| GC74 | Gancao | MOL004988 | Kanzonol F | 32.47 | 0.89 |
| GC75 | Gancao | MOL004989 | 6-prenylated eriodictyol | 39.22 | 0.41 |
| GC76 | Gancao | MOL004990 | 7,2',4'-trihydroxy－5-methoxy-3－arylcoumarin | 83.71 | 0.27 |
| GC77 | Gancao | MOL004991 | 7-Acetoxy-2-methylisoflavone | 38.92 | 0.26 |
| GC78 | Gancao | MOL004993 | 8-prenylated eriodictyol | 53.79 | 0.4 |
| GC79 | Gancao | MOL004996 | gadelaidic acid | 30.7 | 0.2 |
| GC80 | Gancao | MOL000500 | Vestitol | 74.66 | 0.21 |
| GC81 | Gancao | MOL005000 | Gancaonin G | 60.44 | 0.39 |
| GC82 | Gancao | MOL005001 | Gancaonin H | 50.1 | 0.78 |
| GC83 | Gancao | MOL005003 | Licoagrocarpin | 58.81 | 0.58 |
| GC84 | Gancao | MOL005007 | Glyasperins M | 72.67 | 0.59 |
| GC85 | Gancao | MOL005008 | Glycyrrhiza flavonol A | 41.28 | 0.6 |
| GC86 | Gancao | MOL005012 | Licoagroisoflavone | 57.28 | 0.49 |
| GC87 | Gancao | MOL005013 | 18α-hydroxyglycyrrhetic acid | 41.16 | 0.71 |
| GC88 | Gancao | MOL005016 | Odoratin | 49.95 | 0.3 |
| GC89 | Gancao | MOL005017 | Phaseol | 78.77 | 0.58 |
| GC90 | Gancao | MOL005018 | Xambioona | 54.85 | 0.87 |
| GC91 | Gancao | MOL005020 | dehydroglyasperins C | 53.82 | 0.37 |
| GC92 | Gancao | MOL000098 | quercetin | 46.43 | 0.28 |
