# Supplementary material for: Investigation on the mechanism of Shaoyao-Gancao Decoction in the treatment of gastric carcinoma based on network pharmacology and experimental verification
Source: Aging (Albany NY). 2023 Jan 3;15(1):148–63. doi: 10.18632/aging.204465 (PMC9876642; doi:10.18632/aging.204465)
Supplement: Supplementary Table 2 [file aging-15-204465-s002.docx]

**Supplementary Table 2. The molecule name of ingredients.**

| **Number** | **Source** | **Molecule Name** |
| --- | --- | --- |
| SY03 | Shaoyao | beta-sitosterol |
| SY05 | Shaoyao | (3S,5R,8R,9R,10S,14S)-3,17-dihydroxy-4,4,8,10,14-pentamethyl-2,3,5,6,7,9-hexahydro-1H-cyclopenta[a]phenanthrene-15,16-dione |
| SY07 | Shaoyao | paeoniflorin |
| GC01 | Gancao | Inermine |
| GC02 | Gancao | DFV |
| M1 | Shaoyao/Gancao | Mairin |
| GC04 | Gancao | Glycyrol |
| GC05 | Gancao | Jaranol |
| GC06 | Gancao | Medicarpin |
| GC07 | Gancao | isorhamnetin |
| S1 | Shaoyao/Gancao | sitosterol |
| GC09 | Gancao | Lupiwighteone |
| GC10 | Gancao | 7-Methoxy-2-methyl isoflavone |
| GC11 | Gancao | formononetin |
| GC12 | Gancao | Calycosin |
| K1 | Shaoyao/Gancao | kaempferol |
| GC14 | Gancao | naringenin |
| GC15 | Gancao | (2S)-2-[4-hydroxy-3-(3-methylbut-2-enyl)phenyl]-8,8-dimethyl-2,3-dihydropyrano[2,3-f]chromen-4-one |
| GC16 | Gancao | euchrenone |
| GC17 | Gancao | glyasperin B |
| GC18 | Gancao | glyasperin F |
| GC19 | Gancao | Glyasperin C |
| GC20 | Gancao | Isotrifoliol |
| GC21 | Gancao | (E)-1-(2,4-dihydroxyphenyl)-3-(2,2-dimethylchromen-6-yl)prop-2-en-1-one |
| GC22 | Gancao | kanzonols W |
| GC23 | Gancao | (2S)-6-(2,4-dihydroxyphenyl)-2-(2-hydroxypropan-2-yl)-4-methoxy-2,3-dihydrofuro[3,2-g]chromen-7-one |
| GC24 | Gancao | Semilicoisoflavone B |
| GC25 | Gancao | Glepidotin A |
| GC26 | Gancao | Glepidotin B |
| GC27 | Gancao | Phaseolinisoflavan |
| GC28 | Gancao | Glypallichalcone |
| GC29 | Gancao | 8-(6-hydroxy-2-benzofuranyl)-2,2-dimethyl-5-chromenol |
| GC30 | Gancao | Licochalcone B |
| GC31 | Gancao | licochalcone G |
| GC32 | Gancao | 3-(2,4-dihydroxyphenyl)-8-(1,1-dimethylprop-2-enyl)-7-hydroxy-5-methoxy-coumarin |
| GC33 | Gancao | Licoricone |
| GC34 | Gancao | Gancaonin A |
| GC35 | Gancao | Gancaonin B |
| GC36 | Gancao | licorice glycoside E |
| GC37 | Gancao | 3-(3,4-dihydroxyphenyl)-5,7-dihydroxy-8-(3-methylbut-2-enyl)chromone |
| GC38 | Gancao | 5,7-dihydroxy-3-(4-methoxyphenyl)-8-(3-methylbut-2-enyl)chromone |
| GC39 | Gancao | 2-(3,4-dihydroxyphenyl)-5,7-dihydroxy-6-(3-methylbut-2-enyl)chromone |
| GC40 | Gancao | Glycyrin |
| GC41 | Gancao | Licocoumarone |
| GC42 | Gancao | Licoisoflavone |
| GC43 | Gancao | Licoisoflavone B |
| GC44 | Gancao | licoisoflavanone |
| GC45 | Gancao | shinpterocarpin |
| GC46 | Gancao | (E)-3-[3,4-dihydroxy-5-(3-methylbut-2-enyl)phenyl]-1-(2,4-dihydroxyphenyl)prop-2-en-1-one |
| GC47 | Gancao | liquiritin |
| GC48 | Gancao | licopyranocoumarin |
| GC49 | Gancao | 3,22-Dihydroxy-11-oxo-delta(12)-oleanene-27-alpha-methoxycarbonyl-29-oic acid |
| GC50 | Gancao | Glyzaglabrin |
| GC51 | Gancao | Glabridin |
| GC52 | Gancao | Glabranin |
| GC53 | Gancao | Glabrene |
| GC54 | Gancao | Glabrone |
| GC55 | Gancao | 1,3-dihydroxy-9-methoxy-6-benzofurano[3,2-c]chromenone |
| GC56 | Gancao | 1,3-dihydroxy-8,9-dimethoxy-6-benzofurano[3,2-c]chromenone |
| GC57 | Gancao | Eurycarpin A |
| GC58 | Gancao | glycyroside |
| GC59 | Gancao | (-)-Medicocarpin |
| GC60 | Gancao | Sigmoidin-B |
| GC61 | Gancao | (2R)-7-hydroxy-2-(4-hydroxyphenyl)chroman-4-one |
| GC62 | Gancao | (2S)-7-hydroxy-2-(4-hydroxyphenyl)-8-(3-methylbut-2-enyl)chroman-4-one |
| GC63 | Gancao | Isoglycyrol |
| GC64 | Gancao | Isolicoflavonol |
| GC65 | Gancao | HMO |
| GC66 | Gancao | 1-Methoxyphaseollidin |
| GC67 | Gancao | Quercetin der. |
| GC68 | Gancao | 3'-Hydroxy-4'-O-Methylglabridin |
| GC69 | Gancao | licochalcone a |
| GC70 | Gancao | 3'-Methoxyglabridin |
| GC71 | Gancao | 2-[(3R)-8,8-dimethyl-3,4-dihydro-2H-pyrano[6,5-f]chromen-3-yl]-5-methoxyphenol |
| GC72 | Gancao | Inflacoumarin A |
| GC73 | Gancao | icos-5-enoic acid |
| GC74 | Gancao | Kanzonol F |
| GC75 | Gancao | 6-prenylated eriodictyol |
| GC76 | Gancao | 7,2',4'-trihydroxy－5-methoxy-3－arylcoumarin |
| GC77 | Gancao | 7-Acetoxy-2-methylisoflavone |
| GC78 | Gancao | 8-prenylated eriodictyol |
| GC79 | Gancao | gadelaidic acid |
| GC80 | Gancao | Vestitol |
| GC81 | Gancao | Gancaonin G |
| GC82 | Gancao | Gancaonin H |
| GC83 | Gancao | Licoagrocarpin |
| GC84 | Gancao | Glyasperins M |
| GC85 | Gancao | Glycyrrhiza flavonol A |
| GC86 | Gancao | Licoagroisoflavone |
| GC87 | Gancao | 18α-hydroxyglycyrrhetic acid |
| GC88 | Gancao | Odoratin |
| GC89 | Gancao | Phaseol |
| GC90 | Gancao | Xambioona |
| GC91 | Gancao | dehydroglyasperins C |
| GC92 | Gancao | quercetin |
